# *QuickStats:* Percentage* of Adults Aged ≥20 Years Who Had Taken Any Dietary Supplement^†^ in the Past 30 Days, by Sex and Family Income^§^ — National Health and Nutrition Examination Survey, United States, 2017–2018

**DOI:** 10.15585/mmwr.mm7001a7

**Published:** 2021-01-08

**Figure:**
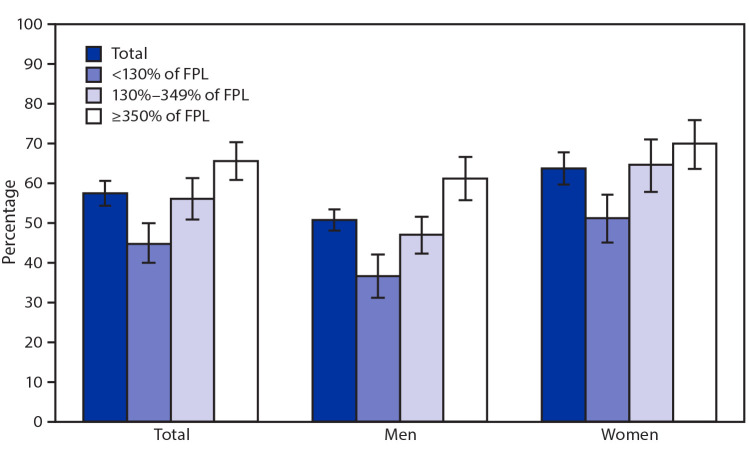
During 2017–2018, 57.6% of adults aged ≥20 years had taken a dietary supplement within the past 30 days. The percentage increased with family income: 44.9% among those with family incomes <130% of the FPL, 56.2% among those with family incomes 130%–349% of the FPL, and 65.7% among those with family incomes ≥350% of the FPL. The increase with family income was seen for both men and women. Women were more likely than were men to use a dietary supplement overall (63.8% versus 50.8%) and at each income level.

**Source:** National Center for Health Statistics, National Health and Nutrition Examination Survey, 2017–2018. https://wwwn.cdc.gov/nchs/nhanes/search/datapage.aspx?Component=Dietary&CycleBeginYear=2017.

